# Simultaneous Bilateral Transitional Fractures of the Proximal Tibia after Minor Sports Trauma

**DOI:** 10.1155/2013/724802

**Published:** 2013-09-28

**Authors:** Mohamed Omar, Maximilian Petri, Max Ettinger, Sebastian Decker, Christian Krettek, Ralph Gaulke

**Affiliations:** Trauma Department, Hannover Medical School, Carl-Neuberg Straße 1, 30625 Hannover, Germany

## Abstract

We report a very rare case of a 16-year-old healthy athletic boy who sustained simultaneous bilateral transitional fractures of the proximal tibia after kicking a football with his right leg during a soccer game. Following minimal invasive plate osteosynthesis with bridging of the growth plate, the patient recovered rapidly without any growth disturbances.

## 1. Introduction

Epiphyseal fractures of the proximal tibia are rare injuries, accounting for 0.5–3% of all epiphyseal injuries [1–4]. There is an age-dependent fracture pattern. Whereas metaphyseal fractures predominantly occur in prepubescent children, tibial spine or Salter Harris type I or II fractures are common in the age from 10 to 12 years. In adolescence, there is a higher prevalence for Salter-Harris type III or IV fractures [5].

With beginning closure of the proximal tibial growth plate, transitional fractures may occur, representing a special type of injury. The growth plate of the proximal tibia closes asymmetrically from posterior to anterior. In this stage, the anterior portion remains vulnerable for tensile forces of the quadriceps muscle that are transmitted by the patellar tendon on the tibial tubercle. These forces may lead to a separation of the anterior growth plate which can extend posteriorly as fracture either through the epiphysis or metaphysis. Transitional fractures of the proximal tibia are rarely reported [6]. However, there is a cumulating incidence in male obese adolescents after inadequate trauma [7]. In contrast, we report the rare case of bilateral simultaneous transitional fractures of the proximal tibia following in a young healthy athletic boy.

## 2. Case Presentation

A 16-year-old soccer player was admitted to our emergency department with painful swelling of both knees after performing a free kick with his right leg during a soccer match. Immediately after the trauma, he developed pain in both knees, consecutively fell on the ground, and was not able to get up. As he tried to bear weight, he collapsed.

The patient had an athletic constitution. He was playing soccer three times a week in a team that took part in a regional championship. He was 195 cm tall and weighed 82 kg. In the year prior to the trauma, he had grown about 25 cm. Medical history was completely clear. In particular, there was no history of joint or bone pathologies. The patient declined the use of any medications.

On physical examination, there were oedematous swelling and intraarticular effusion of both knees. There was a general tenderness on palpation, predominantly at the tibial tuberosity of both sides with crepitation at the right side. Palpation no revealed any fracture of the patella nor a disruption of the quadriceps or patellar tendon. Quadriceps contraction resulted in movement of the patellae. The knees were held in a semiflexed position. Any attempt of motion provoked severe pain. Evaluation of the cruciate and collateral ligaments was therefore limited; however, it did not reveal obvious instabilities. The right lower leg was significantly thickened compared to the opposite side, indicating a developing compartment syndrome. Neurological examination was normal. Pedal pulses were easily palpable.

**Figure 1:**
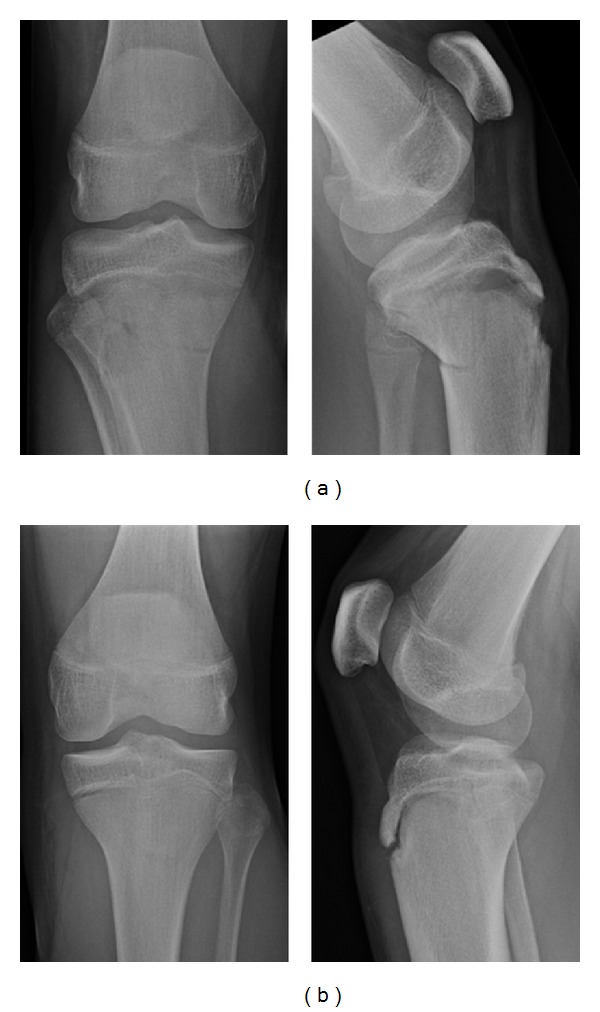
Plain radiographies of the right (a) and left knee (b) after trauma revealed transitional fractures of the proximal tibia, on the right side dorsally displaced, on the left side undisplaced.

Plain radiographies showed transitional fractures of both proximal tibiae as illustrated in Figure 1. The fracture pattern was similar on both sides. There was a gap at the anterior part of the growth plate which extended as a fracture through the dorsal metaphysis, thus creating two large fragments. The posterior part of the growth plate was obviously closed. On the right side, the proximal fragment was displaced as flexion type fracture. On the left side, there was an increased gap in the anterior growth plate either. However, the fracture through the metaphysis remained undisplaced.

**Figure 2:**
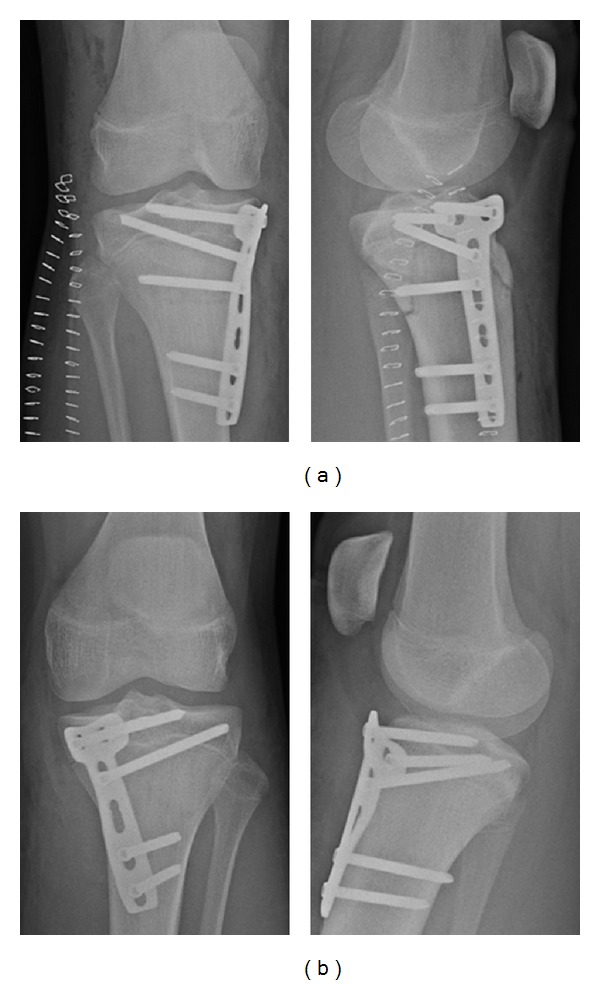
Plain radiographies of the right (a) and left knee (b) after closed reduction and MIPO using two 4.5 mm LCP t-plates placed at the anteromedial aspect of the tibia. Growth plates were bridged.

Due to the developing compartment syndrome, surgery was initiated immediately. Following complete fasciotomy of the right lower leg, we performed a closed reduction and internal fixation by minimal invasive plate osteosynthesis (MIPO) of both proximal tibiae (Figure 2). We decided to bridge the growth plates since they were already partially closed and therefore growth disturbances were not expected. Two 4.5 mm locking compression t-plates (LCP, Synthes, Switzerland) were placed in a minimally invasive technique at the anteromedial aspect of the tibia. Evaluation of the cruciate and collateral ligaments after osteosynthesis in general anaesthesia did not reveal any instability. The wound of the fasciotomy of the right lower leg was temporarily closed with a vacuum sealed drainage. After soft tissue consolidation, we removed the drainage and performed a secondary wound closure five days later.

Postoperatively, we restricted weight bearing on both sides for two weeks; thus, the patient was mobilized in a wheelchair. Subsequently, we allowed full weight bearing of the left leg and partial weight bearing of the right leg for four weeks. There was no restriction for range of motion at any time. Plain radiographies after six weeks showed nearly complete consolidation of the fractures. Therefore, we continued mobilization with full weight bearing. 

**Figure 3:**
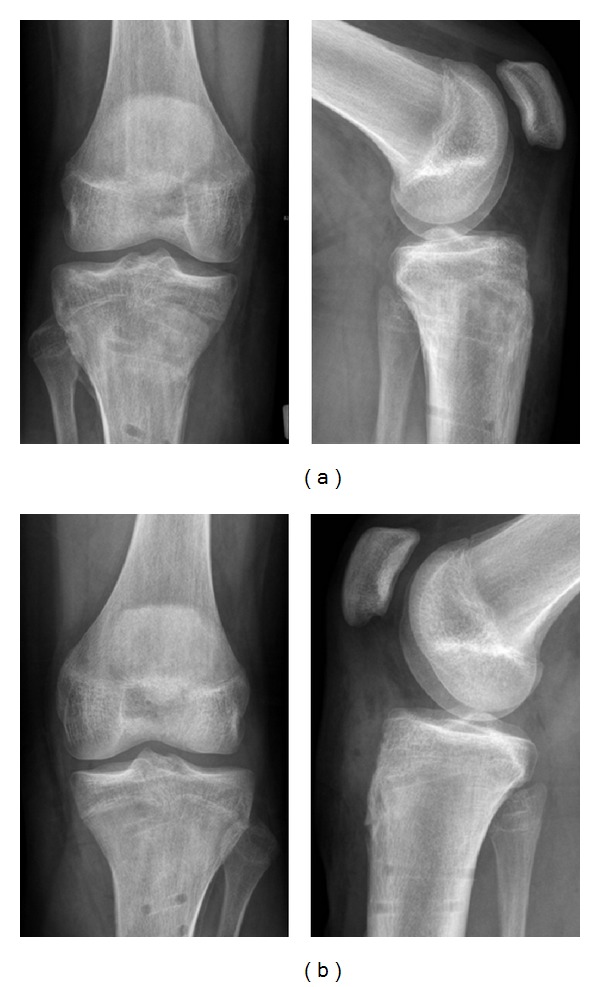
Plain radiographies of the right (a) and left knee (b) following implant removal four months after trauma. Bony healing in anatomic position, growth plates fused.

**Figure 4:**
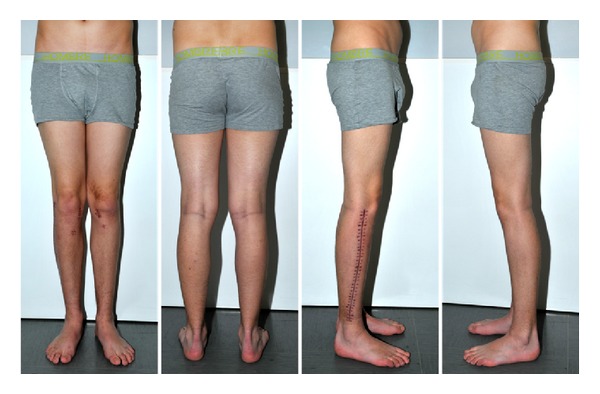
After implant removal, leg axis was normal and leg length equal.

**Figure 5:**
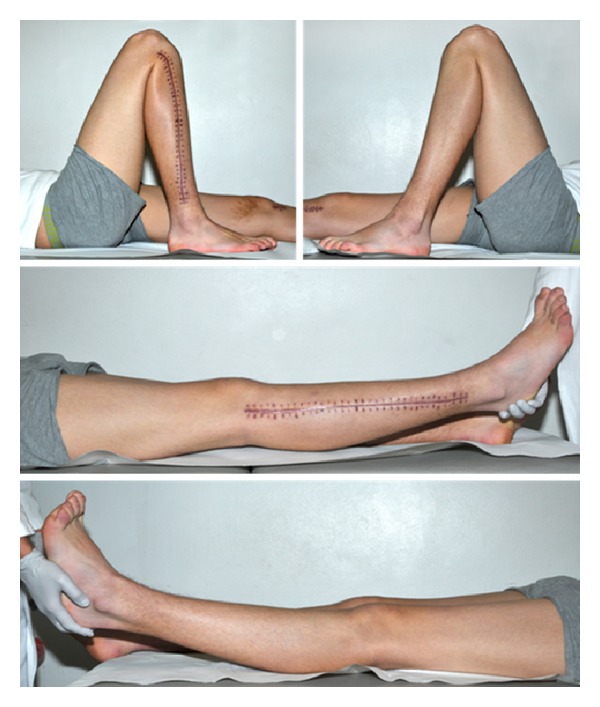
After implant removal, ROM of the knee was 0/0/150° on the right side and 10/0/135° on the left side.

Four months after trauma, we removed the implants since fractures were consolidated completely and growth plates were closed (Figure 3). The patient had no limitations in daily routine and started playing soccer again. There were no signs of posttraumatic growth disorder. Alignment and axis of the knee joints were symmetrical. Leg length was equal (Figure 4). There was no knee instability. Range of motion of the knee was 0/0/150° on the right side and 10/0/135° on the left side (Figure 5).

## 3. Discussion

While fractures of the infantile and adolescent distal tibia are common [8], the proximal tibia is rarely involved. Reported incidences vary from 0.5% to 3% of all epiphyseal fractures [1–4]. In contrast to the distal tibia, the proximal tibia has a high intrinsic stability. The medial collateral ligament is attached at the metaphysis while the lateral side is buttressed by the fibula. Thus, valgus und varus forces are not directly transmitted to the epiphysis. However, tensile forces transmitted through the patellar tendon on the tibial tuberosity may lead to anterior lysis of the growth plate. Among a variety of factors, the complex ossification sequence of the proximal tibial growth plate during maturation leads to an age-dependent injury pattern [5]. The proximal tibia has two ossification centres, namely, the epiphysis and tibial tuberosity which fuse by the fifteenth year of age. Prior to the fusion, avulsion of the tibial tubercle may occur as result of tensile forces transmitted through the quadriceps muscle. Following fusion, these forces predominantly lead to growth plate separations. Complete closure of the a growth plate occurs within 18 months. Ossification starts in the posterior and extends to the anterior portion. During this phase, the growth plate consists of an anterior cartilaginous and posterior osseous part [1]. In this constellation, the anterior part remains vulnerable for tensile forces of the quadriceps muscle. This may lead to a separation of the cartilaginous part of the growth plate that extends as fracture either through the metaphysis or epiphysis. Due to traction of the quadriceps muscle, the proximal fragment is typically displaced in a flexed position with dorsal displacement of the epiphysis [9]. In contrast, prior to closure of the growth plate, injuries of the proximal tibia lead to displacement of the metaphysis, while the epiphysis remains in a constant relation with the distal femur [5, 10, 11].

Along this line, our patient had a separation of the anterior epiphysis which extended as fracture through the dorsal metaphysis. The trauma mechanism was typical for flexion type injuries created by tensile forces of the quadriceps muscle. The performance of a free kick involves a complex chain of motion with contraction of the quadriceps muscles in both the kicking right leg and the supporting left leg. As a consequence, higher tensile forces in the kicking right leg led to a displacement of the epiphysis. Frequently, this type of fracture is the result of an indirect trauma involving eccentric contraction of the quadriceps muscle on a flexed knee such as kicking a ball or commencing a jump. As discussed earlier, dependent on skeletal maturity, this trauma results either in an avulsion of the tibial tubercle or a separation of the growth plate [12]. The concomitant development of a compartment syndrome after proximal tibial fractures is a common side effect which can be explained by affection of branches of the anterior tibial recurrent artery [13].

Injuries of the proximal tibial epiphysis typically occur in male obese children with features of adrenogenital syndrome. Hormonal changes during puberty and consecutive loosening of the cartilage of the growth plate were suspected as causing factors. The exact pathological background remains unclear [14]. In contrast, our patient had an athletic constitution and no history for joint or bone pathologies. He did not suffer from any medical condition and had no medication. The exclusive risk factor was his growth of 25 cm within a year prior to the trauma, possibly leading to a weakening of the growth plate [15]. During the last three months, he reported that there was no further growth which is in line with the radiographic signs of beginning ossification of the dorsal growth plate.

Although epiphyseal fractures of the proximal tibia are rarely reported in children and adolescents, there is a cumulating incidence for bilateral occurrence. Physiological changes during preparation for closure may be predictive of epiphyseal separation. Both consecutive and simultaneous fractures have been described so far. Reported fracture types include consecutive Salter-Harris type I and II [16], type II [17] type I, and transitional type [6] as well as simultaneous type I and II [18], type II [19, 20], type II and III [7]. Bilateral simultaneous transitional fractures of the proximal tibia have not been described yet. However, reevaluation of the reported cases indicates that these fractures may easily be misinterpreted as Salter Harris type II injury [7, 19]. 

Strict differentiation of transitional fractures and fractures of the open epiphysis is strongly recommended. Since the partially closed epiphysis is an indicator for high skeletal maturity, limb-length discrepancies and angular deformities are not expected posttraumatically [8]. In turn, this has consequences on the choice of therapy. In contrast to epiphyseal fractures with completely opened growth plates, transitional fractures can be handled like proximal tibial fractures in adults. Therefore, we decided in our case to perform a plate osteosynthesis with bridging of the growth plate. This enabled a good primary stability and allowed an early functional postoperative treatment with full recovery after three months. 

Closed reduction and fixation with K-wires or conservative treatment of transitional fractures as performed by other authors bears several disadvantages. Besides long immobilisation and reduced stability, refracturing may occur easily [19]. Since no significant growth is expected in patients with beginning closure of the growth plate, the aim should be anatomical reduction and high primary stability of the osteosynthesis instead of preservation of an open growth plate.

To summarize, open reduction and plate osteosynthesis with bridging of the growth plate can yield excellent results with high primary stability for patients with transitional fractures of the proximal tibia and should be considered as primary treatment.
